# Supplementary material for: Label-Free, Rapid and Facile Gold-Nanoparticles-Based Assay as a Potential Spectroscopic Tool for Trastuzumab Quantification
Source: Nanomaterials (Basel). 2021 Nov 24;11(12):3181. doi: 10.3390/nano11123181 (PMC8708960; doi:10.3390/nano11123181)
Supplement: Supplementary file 1 [file nanomaterials-11-03181-s001.zip › nanomaterials-1433893-supplementary.pdf]

Supporting Information

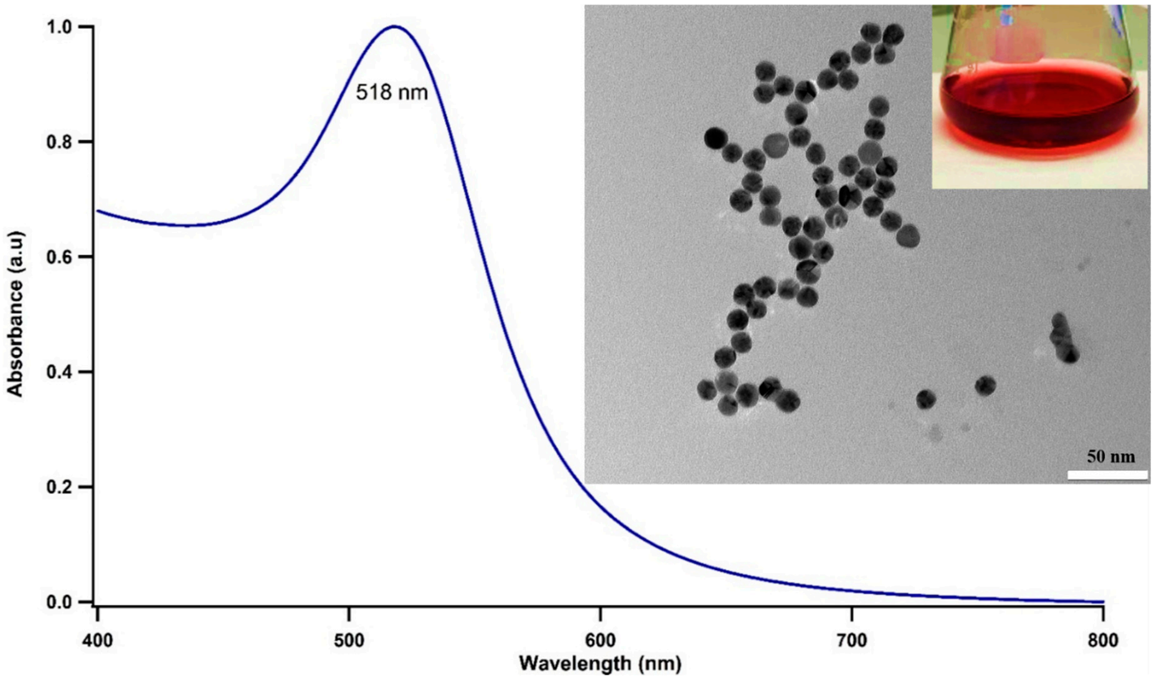

Figure S1. UV-Vis spectrum of AuNPs synthesized using citrate reduction method and the corresponding TEM micrograph. Red color of the solution confirmed the production of AuNPs.

SI.2 Assessment of HER2-Trastuzumab recognition via indirect ELISA

Indirect ELISA was performed to confirm HER2-Trastuzumab binding. Firstly, the wells (96-well plate) were coated with 1 and 5 ng/µl of Trastuzumab (100 mM sodium carbonate/bicarbonate, pH 9.6) and incubated overnight at 4°C. The following day, the wells were washed 3x, with PBST (PBS +Tween 0.05%), and incubated for an hour with a blocking solution (PBST+1% BSA) followed by 4x of washings. Next, His-HER2 (5, 25, and 50 ng) protein was added to the wells and left to react for 3 hours. The plates were washed 4x, 5 min each. 100 µl of the antibody anti-His (HRP Sigma #A7058) was diluted 1:2000 in PBST+1%BSA and was added to the wells, incubated for 90 min, and followed by 4x washings for 5min each. Finally, 100 µl TMB was added to each well and incubated

for 15 min. The reaction was stopped with the addition of 1.0 M HCl. The absorption of each well was determined at 450 nm by an ELISA plate reader (Tecan, Infinite F200 Pro). The experiment was carried out in duplicate. ELISA results reported in **Figure S2** confirmed the ability of the Trastuzumab to recognize His-HER2 protein receptor. The findings indicated that 50 ng of immobilized Trastuzumab can bind His-HER2 in a linear range of 0.5-5 ng/ml of the antigen.

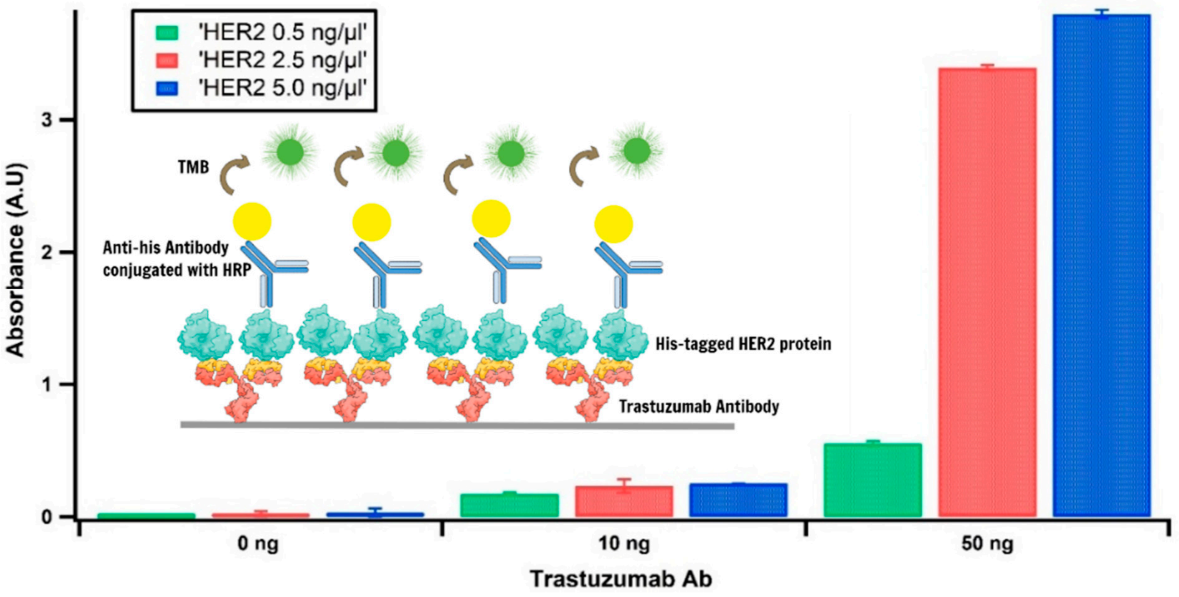

Figure S2. Indirect ELISA analysis performed for Trastuzumab antibody with various concentrations of His-HER2 antigen

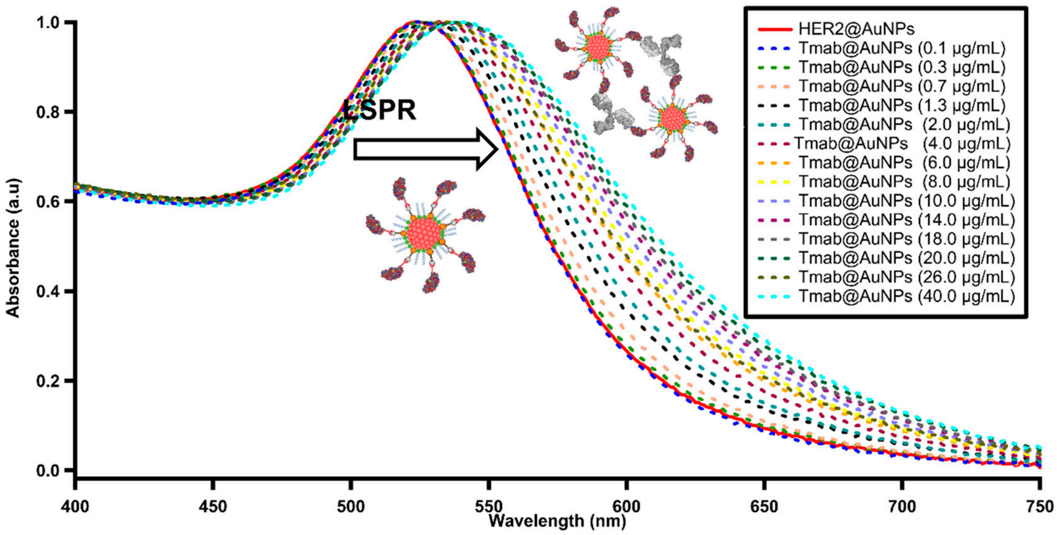

Figure S3. UV-Vis absorption spectra for His-HER2 decorated AuNPs before and after the incubation with Trastuzumab. LSPR peaks redshifted upon varying the concentration of the antibody.

S1.2 Transmission and Scanning Electron Microscopy measurements

TEM measurements were performed on a Philips EM208 electron microscope operating at 100kV. Samples were prepared by casting 5 microliters of solution on 200 mesh carbon coated copper grids. Scanning Electron Microscopy (SEM) images were acquired with a ZEISS FEG-SEM Supra 40. Imaging was performed at 5-10 keV of acceleration voltage by detecting electrons with an Everhart-Thornley Secondary Electron Detector and a High efficiency In-lens Detector. For the preparation of the SEM sample, 0.5-1 cm pre-cut pieces of Si (111) wafer were cleaned by sonication in acetone and isopropanol. Then, a drop of Poly-Ornithine (Sigma-Aldrich) was added, to facilitate the capture of nanoparticles via electrostatic charges. After removing the excess of poly-ornithine by a thorough washing step with Milli-Q water, we deposit 15 µL of nanoparticles and incubated for 15 minutes. The sample was then washed with Milli-Q water, dried with a gentle nitrogen stream and promptly inserted in the SEM chamber for measurements.

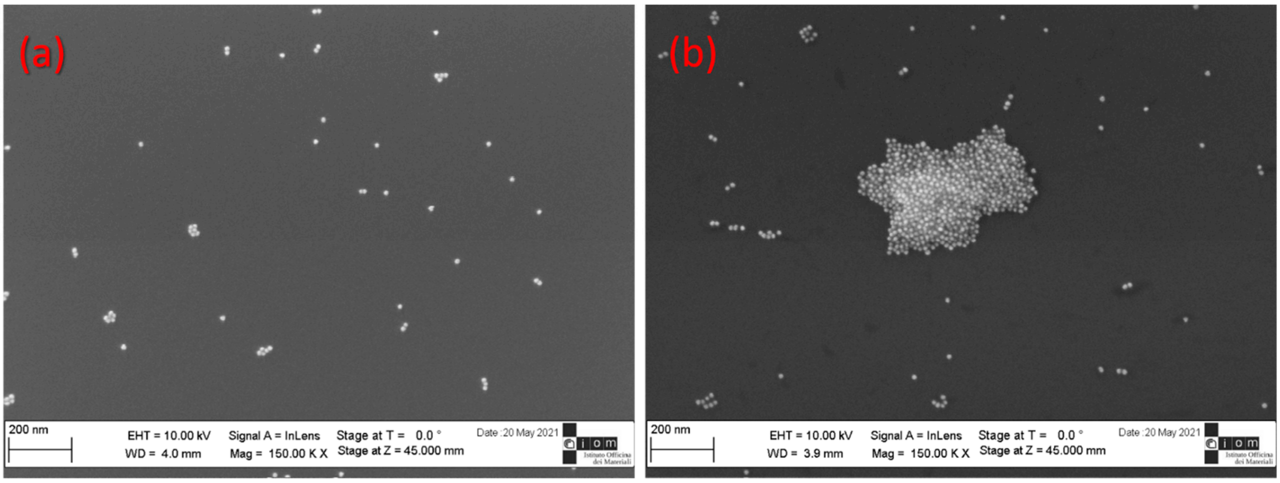

Figure S4. SEM micrographs of (**a**) NTA-TOEG3 coated AuNPs, (**b**) HER2-decorated AuNPs treated with 10 µg/mL Trastuzumab. Note cross-linking of the nanoparticles caused by the addition of the target antibody.

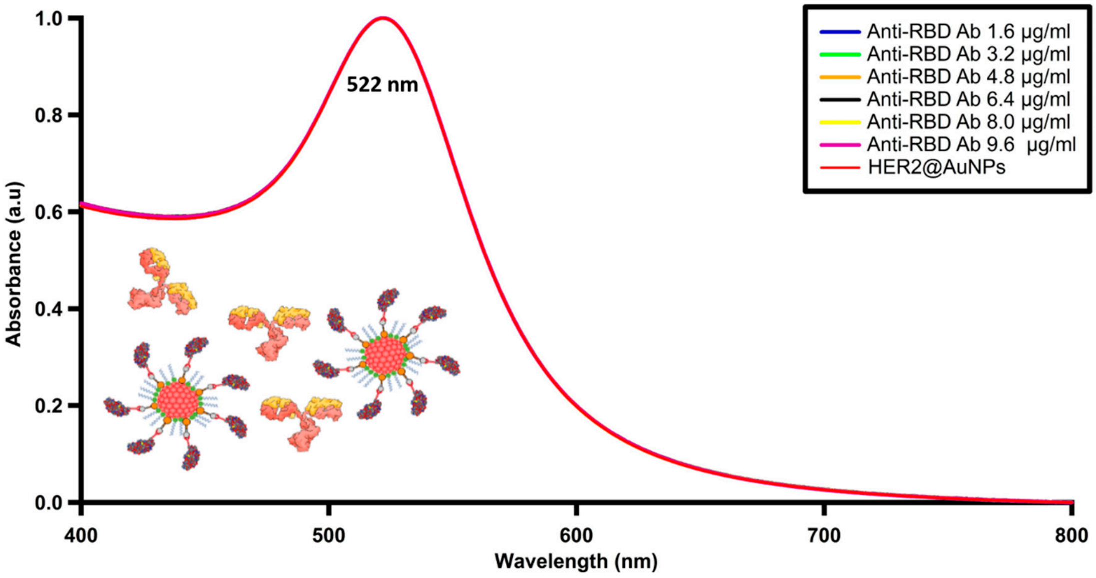

Figure S5. UV-Vis absorption spectra for His-HER2 incubated with various concentrations of non-HER2 recognizing antibody. The spectroscopic signal was similar to HER2 capped AuNPs.

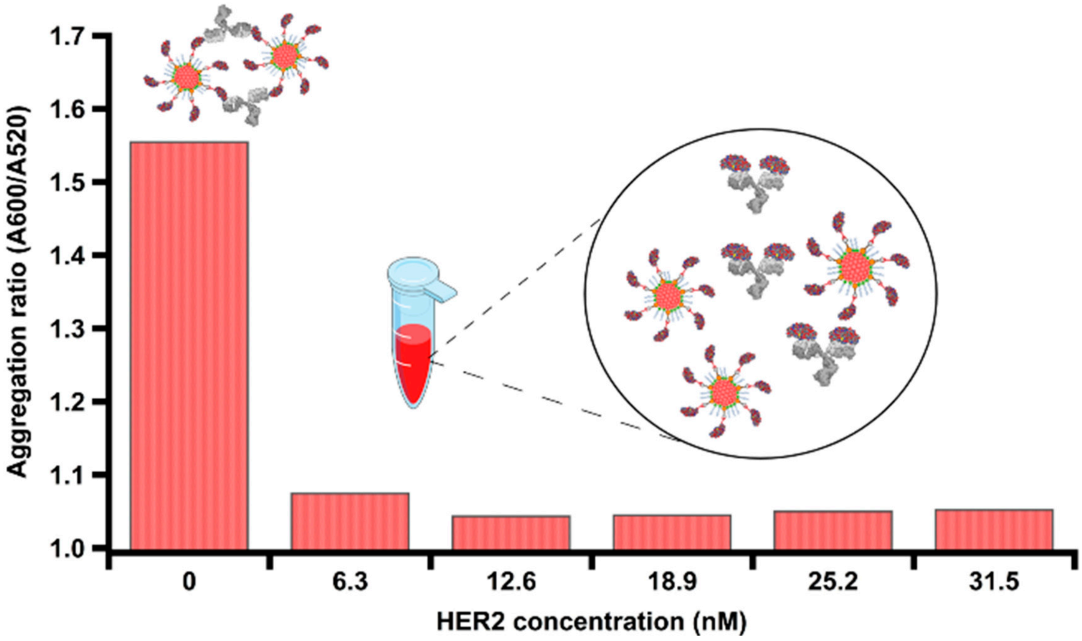

Figure S6. Spectrophotometric titration ranging from 6-32 nM free His-tagged HER2 concentrations.

S1.3 DLS measurements for adjusted SAM density on AuNP

DLS measurements were performed with a Malvern Zetasizer Nano ZS instrument. For particle size determination, 500 μL aliquots of solution were placed in disposable polystyrene cuvettes and analyzed at 25 ºC. The samples were equilibrated for 2 min before analysis, and 3 runs per sample were taken. We studied varying surface molar ratios of NTA and TOEG3. We found that although higher levels of NTA increased LSPR response over all the tested concentrations of the antibody, it revealed a higher degree of aggregation compared to AuNPs capped with lower concentrations of NTA.

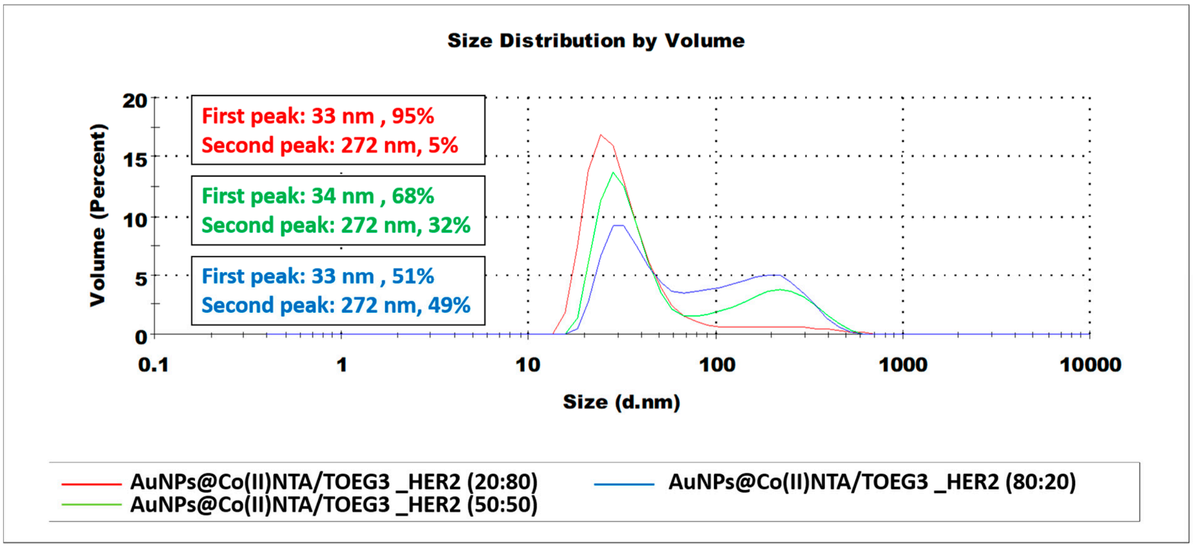

Figure S7. DLS volume-weighted size distributions comparing AuNPs coated with NTA and TOEG3 SAM with molar ratios of 20:80, 50:50, and 80:20, respectively.

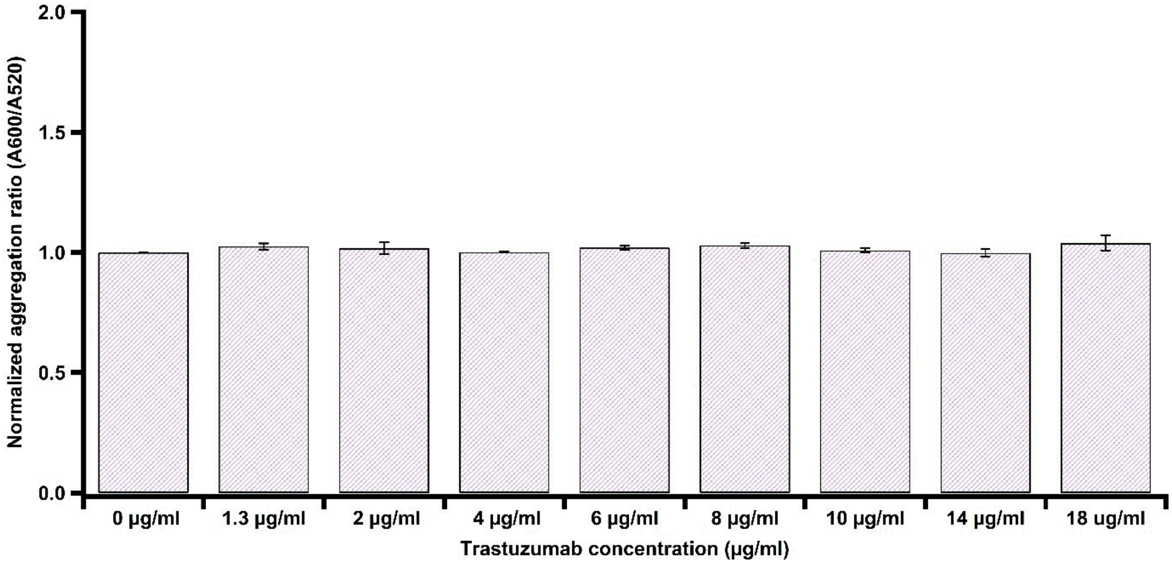

Figure S8. The presence of competing Histidine rich proteins that may have interfered and disrupted the bioconjugate leading to the less sensitive complex formation toward the target antibody.

S1.4 Filtering Human serum from histidine-rich proteins using Ni-NTA agarose matrix

Ni-NTA agarose matrix purchased from QIAGEN (Hilden, Germany) was used to remove proteins containing Histidine-repeats (from 3-10 amino acids). Briefly, 100 µl of the resin was incubated with 900 µl, 5% diluted serum for 30 min, Then, the supernatant was collected after each centrifugation (500×g, 3 min). After the third wash, the matrix was eluted (2M imidazole, pH 8.0). All the samples obtained from each step were run in SDS-PAGE gel (gradient gel).

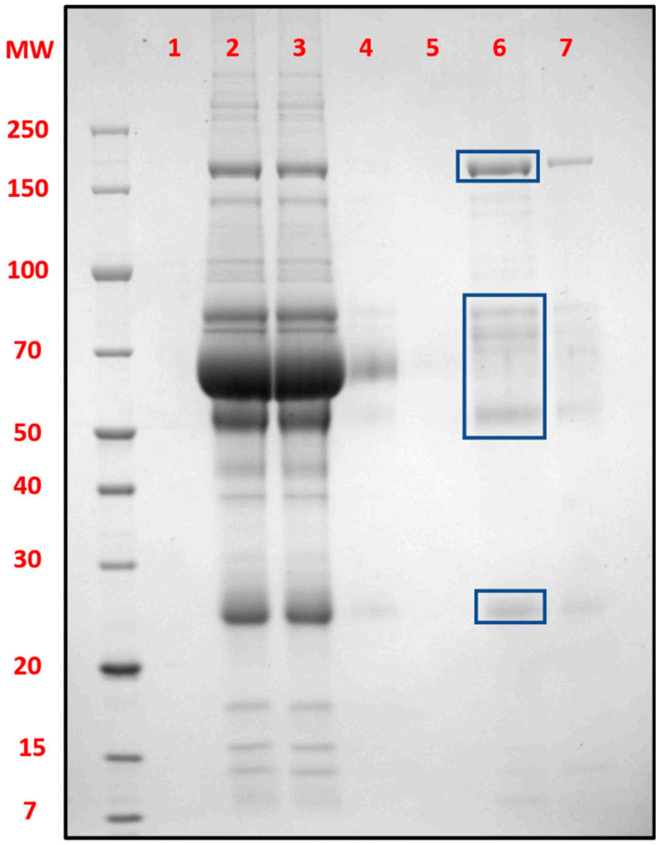

Figure S9. SDS-PAGE image of **(1)** empty lane, (**2**) 5% diluted serum, (**3**) flow through of the serum after incubation with the matrix, (**4**,**5**) first and second wash of the matrix, (**6**) after eluting the Ni-NTA matrix with imidazole, (**7**) the Ni-NTA matrix. Note that proteins containing Histidine-repeats were released after the elution step.

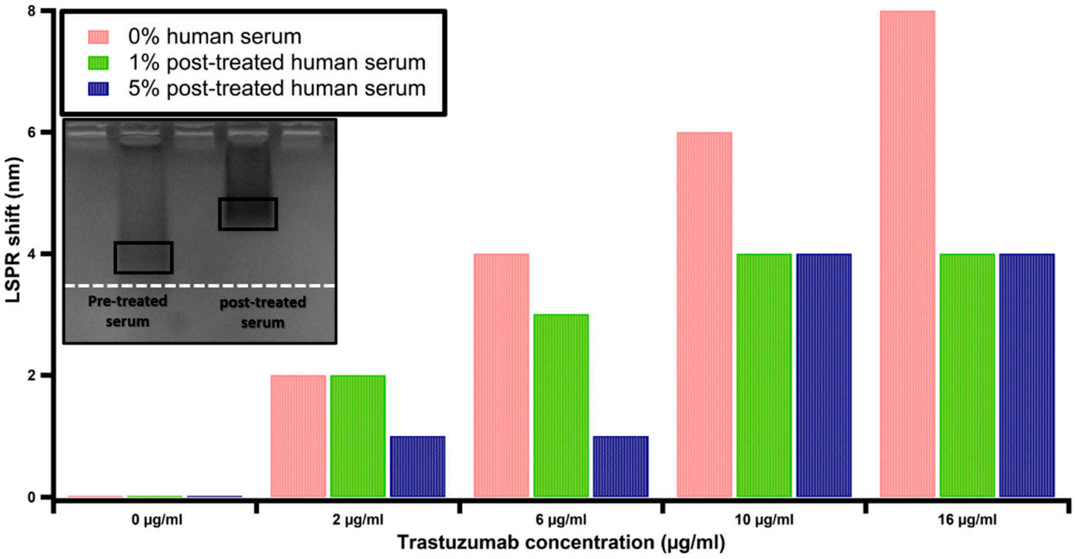

Figure S10. LSPR shifts for the particles treated with various concentration of the target antibody dispersed in 1% and 5% filtered serum. The gel electrophoresis photograph revealed a slower mobility for the immunocomplex hosted by filtered diluted serum.
